# Mental Health, Recovery, and the Community

**DOI:** 10.1155/2013/926174

**Published:** 2013-03-21

**Authors:** Wouter Vanderplasschen, Richard C. Rapp, Steve Pearce, Stijn Vandevelde, Eric Broekaert

**Affiliations:** ^1^Department of Orthopedagogics, Ghent University, H. Dunantlaan 2, 9000 Ghent, Belgium; ^2^Center for Interventions, Treatment and Addictions Research (CITAR), Boonshoft School of Medicine, Wright State University, 3640 Colonel Glenn Highway, Dayton, OH 45435, USA; ^3^Oxfordshire Complex Needs Service, Oxfordshire and Buckinghamshire Mental Health NHS Foundation Trust, Manzil Way, Oxford OX4 1XE, UK; ^4^Faculty of Education, Health and Social Work, Ghent University College, Voskenslaan 362, B-9000 Ghent, Belgium

## 1. Introduction 

The prevalence of mental health problems is high and causes profound health, social, cultural, and economic problems worldwide [1, 2]. Most psychiatric disorders are characterized by a chronic and complex nature and recurring episodes of acute symptoms. For decades, the treatment of mental health problems was primarily situated in residential services. Criticism of the so-called “total institutions” led to the downsizing or even the total close of institutions in favor of community mental health care [3]. Still, the number of psychiatric beds and hospitalizations remains high in several countries. Moreover, the treatment of mental health problems has traditionally been guided by a cure-oriented approach followed by rehabilitation efforts to reinsert individuals in society after substantial periods of hospitalization.

In reaction against what is perceived to be an overly narrow biomedical model, the recovery movement emphasizes the importance of a client-centred approach, based on individuals' perceived needs and goal-directed practices that reflect clients' valued activities [4]. Instead of rehabilitation, in which clients' role in regaining control over their life is often neglected, recovery focuses on the question how individuals can have more active control over their lives (agency). Recovery has been defined as “a deeply personal, unique process of changing one's attitudes, values, feelings, goals, skills, and roles. It is a way of living a satisfying, hopeful, and contributing life, even with any limitations caused by illness” [5, 6]. It is characterised by a search for the person's strengths and capacities, satisfying and meaningful social roles, and mobilizing formal and informal support systems. Recovery has begun to have an influence in thinking more broadly about mental health care and how social inclusion can be promoted [4].

Research on recovery among various mental health populations and on effective strategies and interventions to promote recovery is still in its infancy [6]. Empowerment, hope, responsibility, peer support, advocacy, and quality of life have become predominant concepts in the recovery debate but remain poorly understood from a consumer's perspective. Treatment providers and key workers need to rethink their role as supporters of service users' personal recovery and require the skills and competences necessary for doing so. On the other hand, service users are considered to be the primary agents of their recovery process and need to employ personal and community resources for living a satisfying life. 

In this special issue, we present 11 original papers on the emerging topic of recovery from mental health problems and the role of community services and support. The contributions concern diverse mental health populations, including people with psychosis or schizophrenia, substance use disorders, offenders with mental disorders, and young adults with posttraumatic stress disorders. We focus on formal as well as informal systems for supporting recovery. Empirical data for this special issue were collected using quantitative as well as qualitative research methods and involve diverse stakeholders, including service users themselves. The papers represent contributions from various continents, illustrating the topicality and international relevance of recovery in the field of mental health care.

Three areas of recovery research can be distinguished in this special issue. The first area addresses the conceptualization of recovery and how professionals, relatives, and society at large can be educated regarding the process of recovery from mental health problems. The second area of papers explores the lived experiences of individuals with mental health difficulties and the personal and community resources they employ to enhance their recovery process. The last area focuses on formal and informal support systems that may stimulate recovery among diverse mental health populations.

## 2. The Concept of Recovery and Its Spread

Recovery is an increasingly frequently cited term in mental health research, with over 2000 articles included in the Web of Knowledge (Thomson Reuters) mentioning this term in title or abstract during the last 20 years. Although implicitly understood by most stakeholders, conceptual and theoretical inconsistencies are not uncommon and “clinical” and “personal” recovery should be clearly distinguished [6]. The paper by C. Vandekinderen et al. provides a conceptual framework for understanding recovery from a consumer-centered perspective. They distinguish between an individual and social model of recovery: a universal, normative approach of citizenship as opposed to a relational, inclusive approach which enhances hope and belonging and requires alternative support strategies in community mental health care.

Despite the central objective of social inclusion in the recovery discourse, stigmatization of persons with mental health problems has often been reported [7, 8]. It is characterized by a lack of knowledge and false attributions concerning these problems, leading to social exclusion and feelings of guilt and shame. M. Corbière et al. set up a survey among diverse stakeholders in mental health care in Quebec, Canada, to identify strategies to fight prejudices and stigma. The authors argue that disclosure is a crucial factor in the process of destigmatization. Primary strategies to fight stigma identified by mental health care professionals were education and working on recovery and social inclusion, while service users focused on social contacts and person-centered strategies. The effectiveness of an education and training program for changing professionals' knowledge and attitudes about recovery was tested by G. K. M. L. Wilrycx et al., following a recovery-oriented mental health care reform in The Netherlands. The researchers demonstrated that recovery-oriented training can improve mental health care professionals' attitudes towards recovery, but its effectiveness for increasing knowledge was only temporarily. 

## 3. Lived Experiences of Persons with Mental Health Difficulties

The subjective perspectives of service users and their lived experiences play a pivotal role in the personal recovery movement, but their narratives do not necessarily accord with these of mental health professionals and researchers [6]. These divergent views are illustrated in the paper by M. Lambri et al. who performed a needs assessment among 110 persons with psychosis and their support workers living in diverse supported housing settings in an inner-city area of London. This study underscores the importance of addressing individuals' personal and social needs when implementing support services in the community in order to improve service users' quality of life. Moreover, deinstitutionalization may have unintended and adverse consequences like unemployment, crime, and deprivation (in particular in urban areas), which touches upon recent expert opinions that community treatment is not necessarily of high quality [3] nor better than a hospital admission [2, 9]. 

The lived experiences of 20 persons with auditory hallucinations in the Chinese administrative region of Hong Kong are explored in a paper by P. Ng et al., who report several personal and informal strategies to cope with hearing voices. The authors stress the importance of understanding these persons' symptoms through education programs for formal and informal caregivers and the need for adapted, culturally sensitive recovery programs for minority groups.

Quality of life is regarded as an important indicator of recovery and subjective wellbeing [10]. F. Morisse et al. assessed dimensions of quality of life and support needs among family members and support workers of persons with intellectual disabilities and cooccurring mental health problems. The authors suggest the existence of generic dimensions of quality of life, although appropriate support strategies are needed for this specific population.

The role of community support and personal and social capital in the recovery process is addressed in several papers, but takes a central position in the paper by S. Vindevogel et al., in which they explore the perspectives of former child soldiers and their peers to identify sources of resilience and agency among young adults in the aftermath of the armed conflict in Uganda. The findings from this qualitative thematic analysis call for the development of community-based support systems to enhance individuals' capacities as well as the communal sociocultural fabric in these war-affected societies.

Recovery cannot be defined as an outcome or state to attain but should rather be seen as a process and a satisfying way to live one's life [6]. The relapsing nature of this process is illustrated in the paper by C. Colman and F. Vander Laenen who explored the concepts of “recovery” and “desistance” in a sample of 40 recovering drug offenders. They highlight the role of human agency and critical turning points in the life span of “drug addicts” and “criminals”, but emphasize that desistance is hardly attainable without drug abstinence. 

## 4. Formal and Informal Support Systems towards Recovery

The role of formal and informal support systems in promoting recovery is increasingly studied and has been the subject of some recent reviews [11–13]. In this special issue, R. Pratt et al. report on the effectiveness of Wellness Recovery Action Planning (WRAP), a recovery-based self-management approach to improve individuals' mental health and wellbeing. WRAP is provided in the context of self-help and mutual support groups led by facilitators with lived experiences of mental health difficulties. Based on a qualitative evaluation including interviews and focus groups, the authors found a positive impact of this peer-led intervention, in particular in generating hope and empowering individuals to self-manage their recovery process.

Formal interventions for promoting recovery may include community treatment of offenders with mental health problems. The review by C. Wittouck et al. assesses the impact of drug treatment courts, which divert drug offenders to drug and other types of community treatment instead of sending them to prison, on clinical recovery. Although the findings from this review are indecisive, they provide strong arguments to look beyond substance use and legal outcomes in evaluation studies. 

The therapeutic community (TC) for addictions is another example of a formal support system for supporting clinical recovery among drug addicts. Traditionally, TCs have been evaluated from an acute care perspective, whereas a longitudinal or even a career approach is warranted. The review paper by W. Vanderplasschen et al. focuses on the effectiveness of TCs from a recovery-oriented perspective and regards abstinence as a potential resource, but not as a prerequisite, for recovery defined more broadly. This alternative approach sheds new light on the discussion about TC's effectiveness and proposes a shift in focus from socially desirable outcomes (such as drug abstinence and criminal desistance) to more subjective outcome indicators like family involvement, psychological wellbeing, and engaging in meaningful activities [10]. 

*Wouter Vanderplasschen* *Wouter Vanderplasschen* *Richard C. Rapp* *Richard C. Rapp* *Steve Pearce* *Steve Pearce* *Stijn Vandevelde* *Stijn Vandevelde* *Eric Broekaert* *Eric Broekaert*
